# A simple and feasible questionnaire to estimate menstrual blood loss: relationship with hematological and gynecological parameters in young women

**DOI:** 10.1186/1472-6874-14-71

**Published:** 2014-05-30

**Authors:** Laura Toxqui, Ana M Pérez-Granados, Ruth Blanco-Rojo, Ione Wright, M Pilar Vaquero

**Affiliations:** 1 Institute of Food Science, Technology and Nutrition (ICTAN), Spanish National Research Council (CSIC), C/José Antonio Novais 10, 28040 Madrid, Spain

**Keywords:** Menstruation, Score, Questionnaire, Women, Iron deficiency anemia

## Abstract

**Background:**

Menstrual blood loss (MBL) has been shown to be an important determinant in iron status, work performance and well-being. Several methods have been developed to estimate MBL, the standard quantitative method however has limited application in clinical practice as it is expensive and requires women to collect, store and submit their sanitary products for analysis. We therefore aimed to develop a MBL-score based on a questionnaire, and to validate it by several hematological and biochemical parameters in women of childbearing age.

**Methods:**

A total of 165 healthy young women were recruited. Hematological (hematocrit, hemoglobin, erythrocyte, leucocyte and platelet counts) and iron status (serum iron, serum ferritin, serum transferrin, and total iron binding capacity) parameters were analyzed at baseline. Women were asked to fulfill two gynecological questionnaires: a general questionnaire, to inform about the volunteer’s general menstrual characteristics; and a MBL questionnaire, to provide details of the duration of menstruation, number of heavy blood loss days, and number and type of pads and/or tampons used during the heaviest bleeding day, for all consecutive menstrual periods during 16 weeks. A MBL-score was calculated for each period and women, and its reliability determined by the Cronbach’s alpha coefficient. Pearson’s linear correlation tests were performed between blood parameters and the MBL-score. Two clusters were formed according the MBL-score (cluster 1: low MBL and cluster 2: high MBL).

**Results:**

Significant higher MBL-score was observed in women who reported having a history of anemia (p = 0.015), staining the bed at night during menstruation (p < 0.001) and suffering inter-menstrual blood loss (p = 0.044), compared to those who did not. Women who used hormonal contraceptives presented lower MBL-scores than the others (p = 0.004). The MBL-score was negatively associated with log-ferritin (p = 0.006) and platelet count (p = 0.011). Women in cluster 1 presented higher ferritin (p = 0.043) than women in cluster 2.

**Conclusions:**

We developed an easy and practical method for estimating menstrual blood loss based on a score calculated from a questionnaire in healthy women at childbearing age. The MBL-score is highly reliable and reflects menstrual blood loss validated by hematological and biochemical parameters.

## Background

During a woman’s reproductive years, the time between menarche and menopause, menstruation is a natural and physiological monthly process. However, abnormal bleeding intensity, menorrhagia or intracyclic bleeding, substantially decrease women´s quality of life [1-3]. Menorrhagia, which is defined as an excessive uterine bleeding (>80 mL of blood loss per cycle) occurring at regular intervals, or prolonged uterine bleeding lasting more than seven days, is a common clinical problem among women of reproductive age and frequently results in anemia, impairing women’s daily activities, and is often managed by hysterectomy [4].

Iron deficiency anemia, the most common form of anemia all over the world [5,6], has a complex etiology and is the result of a long-term negative iron balance in the body. A number of factors including low dietary iron bioavailability and high iron requirement contribute to this problem. Women at childbearing age constitute an important risk group due to the additional iron demands of menstruation and pregnancy [7,8].

As menstrual blood loss (MBL) has been shown to be an important determinant in iron status, work performance and well-being [3,9], it is important to find a precise and practical method for its measurement. Several methods for menstrual blood loss quantification have been developed: self-estimate of menstrual loss, counting the number of days of menstruation, counting the number of sanitary products, weighing sanitary products, full blood count (based on hemoglobin levels), pictorial blood loss assessment, menstrual pictogram, and chemical analysis of the blood content of used sanitary products, also known as alkaline hematin method [2,10,11]. This method, considered the gold standard, was developed by Hallberg and Nilsson in 1964 and consists of extracting the heme from the used tampons and pads by sodium hydroxide and converting it to alkaline hematin, which is later determined spectrophotometrically [12]. However, it has limited application in clinical practice as it is expensive and requires women to collect, store and submit all their sanitary products from one cycle/period for analysis, which may be burdensome and even unacceptable for some women [10]. Moreover, it has been suggested that up to 12% of menstrual blood could be lost extraneously and is non-quantified [13].

Our research group has experience in studies with iron-deficient women and has previously described that MBL, together with diet and genetics, is an important factor in iron deficiency etiology [14-16]. We therefore aimed to develop an easy and practical method for estimating menstrual blood loss based on a score calculated from a questionnaire and to validate it by several hematological and biochemical parameters in women of childbearing age.

## Methods

### Subjects

Volunteers were recruited by different announcements in press, university campus and web pages of Madrid. The study was also verbally promoted at public events.

Healthy women aged 18–35 years, Caucasian, non-smoker, non-pregnant, non-breastfeeding and non-anemic were recruited. Exclusion criteria were as follows: amenorrhea (lack of menstruation in the 3 months prior to the study), menopause, iron-metabolism-related diseases (iron deficiency anemia, thalassemia and hemochromatosis), chronic gastric diseases (inflammatory bowel disease, Crohn disease, gastric ulcers, celiac disease, hemorrhagic diseases and helicobacter pylori), renal disease or blood donor status.

This study is part of a wider investigation comprising health questionnaires, a genetic study and nutritional intervention. A total of 584 women contacted the research group to receive information and 289 underwent the screening. Women who did not meet the inclusion criteria or declined to participate were excluded and a total of 165 women agreed to participate, completed the study and were analyzed.

The present study was conducted according to the guidelines laid down in the Declaration of Helsinki, and all procedures were approved by the Clinical Research Ethics Committees of Hospital Puerta de Hierro, Majadahonda (#266, 23 May 2011) and Spanish National Research Council (CSIC), Madrid, Spain. All subjects signed informed consent.

### Gynecological questionnaires

**Figure 1:**
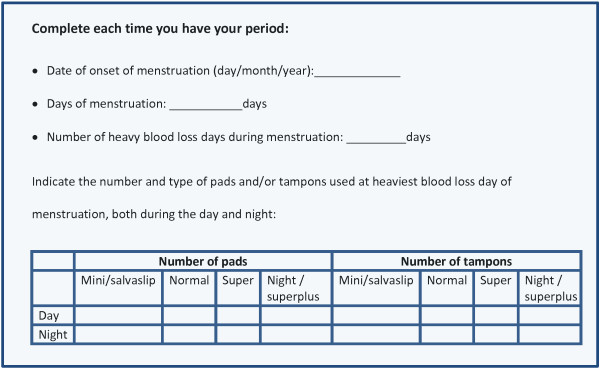
Gynecological questionnaire.

Two questionnaires were used. The first one consisted of the volunteer´s general menstrual characteristics: age at menarche, history of anemia, if they ever stain the bed at night during menstruation, if they ever had inter-menstrual blood loss and the use of any contraceptive method (type, duration and possible change of method in the previous 3 months and during the assay). Women completed this questionnaire at baseline and after 16 weeks in order to track any changes.The second questionnaire was designed to estimate MBL and calculate a score (MBL-score); women were asked the exact start date of menstruation, the duration of menstruation, as well as the number of heavy blood loss days. They were also asked the number and type of pads and/or tampons used during the day and night for the heaviest bleeding day (Figure 1). Women were instructed to fill out this questionnaire for all consecutive menstrual periods during 16 weeks and to do it immediately after using the sanitary products, never from memory.

To estimate the MBL of the volunteers, each kind of pads and tampons were assigned a relative absorbance number according to the manufacturers reported absorbency levels, and multiplied by the number of units used reported by each subject. The absorbance numbers were for pads: mini 1, normal, 1.5, super 2, night/superplus 3; and for tampons: mini 0.5, regular 1, super 1.5, superplus 3.

For example: number of pads reported = 1 (night); number of tampons reported = 4 (super). Therefore, MBL = (1 × 3) + (4 × 1.5) = 9.

The MBL-score was calculated taking into account the duration of menstruation, the number of heavy days and the estimated MBL, see the ‘MBL-score formula’ subsection. This score was calculated for each women and menstrual period.

### MBL-score formula

The MBL-score was calculated as follows:

$$\mathrm{MBL}‒\mathrm{score}\;=\frac{\mathrm{Number}\;\mathrm{of}\;\mathrm{heavy}\;\mathrm{days}}{\mathrm{Number}\;\mathrm{of}\;\mathrm{days}\;\mathrm{of}\;\mathrm{menstruation}}\times \mathrm{MBL}$$

### Anthropometric determinations

All participants were instructed not to deviate from their regular habits and to maintain them during the assay. At baseline, body weight was measured with a Seca scale (to a precision of 100 g) and height was measured with a stadiometer incorporated into the scale. Body mass index (BMI) was calculated. In addition, volunteers completed a general questionnaire every month in order to monitor possible health problems, medication use and changes in their normal routine.

### Blood sampling and hematological and biochemical determinations

All volunteers attended the laboratory facilities at baseline. Blood samples were collected by venipuncture after a 12-h fasting period. Serum was obtained after centrifugation at 1000 g for 15 minutes. Hematocrit, hemoglobin, and erythrocyte, leucocyte and platelet counts were determined using the Symex NE 9100 automated hematology analyzer (Symex, Kobe, Japan). Serum iron, serum ferritin and serum transferrin were determined by the Modular Analytics Serum Work Area analyzer (Roche, Basel, Switzerland). Total iron binding capacity (TIBC) and transferrin saturation were calculated as follows: TIBC (μmol/L) = 25.1 × transferrin (g/L); transferrin saturation = serum iron (μmol/L)/TIBC × 100.

All determinations were subjected to the ISO-9001-2000 requirements.

### Statistical analysis

Data are presented as means with their standard deviations. A normal distribution of variables was determined by the Kolmogorov-Smirnov test and serum ferritin values were log transformed for statistical testing. Reliability of the MBL score was analyzed by the Cronbach’s alpha coefficient. A value >0.70 is usually regarded as satisfactory and the nearer this coefficient is to 1 the more reliable it is [17]. ANOVA was applied to the mean MBL-score, in order to compare women who reported a history of anemia, staining the bed at night during menstruation, inter-menstrual blood loss and the use of a hormonal contraceptive method, with those women who did not. Pearson’s linear correlation tests between MBL-score and hematological and biochemical parameters were performed. Women were grouped according to their MBL-score by means of k-means cluster analysis. Two clusters were formed and differences between clusters were analyzed by ANOVA. Hormonal contraceptive use was analyzed as a random effect factor. A p value of <0.05 was considered significant. The IBM SPSS statistical package for Windows (version 20.0) was used to analyze the data.

## Results

Baseline characteristics of the participating women are shown in Table 1. The Cronbach’s alpha coefficient for the calculated MBL-score was 0.83.

**Table 1:** Baseline characteristics of the women

	**Mean ± SD**
Age (years)	25.3 ± 4.3
Body Mass Index (kg/m^2^)	21.6 ± 3.0
Age at menarche (years)	12.7 ± 1.5
Cycle length (days)	30.2 ± 5.0
Length of period (days)	5.0 ± 1.2
Number of heavy days	1.9 ± 0.8
Menstrual blood loss (units)	8.6 ± 3.5
Total erythrocytes (×10^-12^/L)	4.4 ± 0.3
Hematocrit (%)	39.3 ± 2.6
Mean corpuscular volume (fL)	90.4 ± 4.3
Red blood cell distribution width (%)	13.8 ± 0.9
Hemoglobin (g/dL)	13.3 ± 0.8
MCH (pg)	30.5 ± 1.7
Serum iron (μg/dL)	90.0 ± 41.3
Serum ferritin (ng/mL)	35.2 ± 27.0
Serum transferrin (mg/dL)	291.1 ± 57.0
Transferrin saturation (%)	23.1 ± 12.1
TIBC (μmol/L)	73.1 ± 14.3
Platelets (×10^9^/L)	221.0 ± 44.2
Mean platelet volume (fL)	8.8 ± 1.0
Platelet distribution width (%)	16.2 ± 0.5
Leucocytes (×10^9^/L)	6.6 ± 1.5

MCH, mean corpuscular hemoglobin; TIBC, total iron binding capacity.

A significantly higher MBL-score was observed in women who reported having a history of anemia (p = 0.015), staining the bed at night during menstruation (p < 0.001) and having inter-menstrual blood loss (p = 0.044), compared to those who did not (Table 2).

**Table 2:** Menstrual blood loss score according to gynecological parameters

	**MBL-score**	***p***
History of anemia		
Yes (34%, n = 56)	4.0 ± 2.0	0.015
No	3.2 ± 1.9	
Stain the bed at night during menstruation		
Yes (29%, n = 48)	4.6 ± 1.9	<0.001
No	3.0 ± 1.8	
Inter-menstrual blood loss		
Yes (8.5%, n = 14)	4.5 ± 1.9	0.044
No	3.3 ± 1.9	
Hormonal contraceptive use		
Yes (30.5%, n = 50)	2.8 ± 1.7	0.004
No	3.7 ± 2.0	

MBL, menstrual blood loss.

Percentage of women and n are indicated in parenthesis.

Values are expressed as mean ± SD. Significant differences between groups by ANOVA.

None of the volunteers used intrauterine device as a contraceptive method and 30.5% were using hormonal contraceptives (24.4% contraceptive pills and 6.1% vaginal ring) during the study. It was observed that women who used hormonal contraceptive methods presented lower MBL-score than those who did not (p = 0.004) (Table 2).

The MBL-score was negatively associated with log-ferritin (p = 0.006) and platelet count (p = 0.011). The correlations with the other hematological and biochemical parameters were not significant.

Cluster analysis of the MBL-score formed two different groups (Table 3): cluster 1 (women with low MBL) and cluster 2 (women with high MBL). Serum ferritin and platelet count were significantly lower (p = 0.002 and p = 0.022, respectively) and mean platelet volume significantly higher (p = 0.006) in cluster 2 respect to cluster 1. However, when hormonal contraceptive use was included in the model, differences between clusters in serum ferritin remained significant (p = 0.043) but those in platelet count and platelet volume resulted not significant. The other blood parameters were not significantly different between clusters.

**Table 3:** Iron biomarkers according to cluster score

	**Cluster score**		
	**Low blood loss (n = 117)**	**High blood loss (n = 48)**	***p***
MBL-score	2.4 ± 0.9 (0.5 - 4.2)	6.0 ± 1.4 (4.3 - 10.6)	-
Total erythrocytes (×10^-12^/L)	4,4 ± 0,3	4,3 ± 0,3	NS
Hematocrit (%)	39,4 ± 2,6	39,0 ± 2,4	NS
Mean corpuscular volume (fL)	90,6 ± 4,2	90,0 ± 4,6	NS
Red blood cell distribution width (%)	13,8 ± 1,0	14,0 ± 0,8	NS
Hemoglobin (g/dL)	13,3 ± 0,9	13,1 ± 0,8	NS
MCH (pg)	30,6 ± 1,6	30,3 ± 1,7	NS
Serum iron (μg/dL)	91,6 ± 39,9	86,1 ± 44,9	NS
Serum ferritin (ng/mL)	38,8 ± 27,5	26,2 ± 23,4	0.002*
Serum transferrin (mg/dL)	291,6 ± 57,9	289,8 ± 55,1	NS
Transferrin saturation (%)	23,6 ± 12,2	21,7 ± 11,9	NS
TIBC (μmol/L)	73,2 ± 14,5	72,8 ± 13,9	NS
Platelets (×10^9^/L)	226.0 ± 44,8	208,5 ± 40,6	0.022¶
Mean platelet volume (fL)	8.7 ± 0.1	9.2 ± 0.9	0.006¶
Platelet distribution width (%)	16.2 ± .4935	16.3 ± .5118	NS
Leucocytes (×10^9^/L)	6.7 ± 1.6	6.3 ± 1.4	NS

Values are expressed as mean ± SD (range). MCH, mean corpuscular hemoglobin; TIBC, total iron binding capacity. Significant differences by ANOVA; *p = 0.043 and ¶ NS with hormonal contraceptive use as a random factor.

## Discussion

This study presents an easy and feasible method for estimating menstrual blood losses, based on a score calculated from a questionnaire which is clearly related to history of anemia, several gynecological parameters, hormonal contraceptive use, serum ferritin and platelets, in healthy young women.

The MBL-score is based on three items of the woman's menstrual period that are obtained from a questionnaire that is filled out during menstruation and not from memory. Moreover, the questionnaire was simple and women declared it was easy to complete. Methods based on questionnaires for assessing MBL are reported to be in agreement with the gold standard alkaline hematin method [11,18]. Reliability of the MBL-score was good, according to the scale of Cronbach's alpha coefficients provided by George and Mallery [19]: > 0.9, excellent; > 0.8, good; > 0.7, acceptable, > 0.6, questionable; > 0.5, poor; and < 0.5, unacceptable.

Women, who reported having a history of anemia, staining the bed at night during menstruation or having inter-menstrual blood loss, presented higher scores compared to women who did not report such problems. On the contrary, women who used hormonal contraceptives presented lower MBL-score than those who did not use them. These results support the reasonable accuracy of our score and are in agreement with the literature [20-22]. In addition, the higher the MBL-score the lower the serum ferritin levels which confirms that menstrual bleeding is an important determinant of iron status in women at childbearing age [9]. In this regard, identifying women with high menstrual blood loss could be a key strategy to prevent iron deficiency anemia [7] and to avoid further unnecessary exams.

The fact that women with lower MBL presented higher platelet count, although is in agreement with few case reports in iron deficiency anemic women with menorrhagia [23,24], contrasts with the usual diagnosis of thrombocytosis in iron deficiency anemia [25]. In the present study volunteers were healthy, non-anemic and all their hematological and biochemical values including platelets were within normal ranges and it was observed that hormonal contraceptive use had a confounding influence. In this regard, it is known that hormonal contraceptives increase platelets and the risk of thromboembolism [26].

Therefore, we suggest that the MBL-scores of the present study should be considered as reference values of healthy young menstruating women. The range was between 0.5 and 10.6 and mean values were 2.4 and 6.0, for women with low and high MBL respectively, thus these results could be a starting point in the application of a MBL-score as a risk marker for anemia or other related gynecological disorders associated with menses.

According to Munro [27], our group of women could be classified at normal limits of regularity, duration and frequency of menstrual periods when the 5^th^ to 95^th^ centiles are utilized. Menorrhagia was only observed in 6 women who reported a length of period >7 days. Because the classic definition of menorrhagia (>80 mL of blood loss per cycle) is rarely used clinically due to the difficult to measure menstrual blood loss [4], it could be of interest to apply our questionnaire in women with menorrhagia or other health problems that could affect menstruation, such as malnutrition, anorexia or excessive exercise.

## Conclusions

We have developed an easy and practical method for estimating menstrual blood loss based on a score calculated from a questionnaire in healthy women of childbearing age. It is highly reliable and reflects menstrual blood loss validated by biochemical parameters.

## Abbreviations

BMI: Body Mass Index; MBL: Menstrual Blood Loss; TIBC: Total Iron Binding Capacity.

## Competing interests

The authors declare that they have no conflict of interest.

## Authors’ contributions

The authors’ responsibilities were as follows: LT, AMPG and IW implemented the recruitment and contributed to the analytical determinations and data collection; LT and MPV contributed to the manuscript preparation; LT, AMPG, RBR, IW and MPV contributed to the questionnaire design; and MPV was the principal investigator of the study. All authors have read and approved the final manuscript.

## Pre-publication history

The pre-publication history for this paper can be accessed here:

http://www.biomedcentral.com/1472-6874/14/71/prepub
